# Effect of antibiotic pretreatment on bacterial engraftment after Fecal Microbiota Transplant (FMT) in IBS-D

**DOI:** 10.1080/19490976.2021.2020067

**Published:** 2022-01-11

**Authors:** Prashant Singh, Eric J Alm, John M. Kelley, Vivian Cheng, Mark Smith, Zain Kassam, Judy Nee, Johanna Iturrino, Anthony Lembo

**Affiliations:** a Division of Gastroenterology, Department of Medicine, University of Michigan, Ann, Arbor, Michigan; b Center for Microbiome Informatics and Therapeutics, Massachusetts Institute of Technology, Cambridge, MA, USA; c Department of Psychology, Endicott College, Beverly, MA, USA; d Harvard Medical School, Boston, MA; e Division of Gastroenterology, Beth Israel Deaconess Medical Center and Harvard Medical School, Boston, MA, USA; f Finch Therapeutics, Somerville, MA, USA

**Keywords:** Microbiome, dysbiosis, fecal therapy, fecal microbiota transfer

## Abstract

Fecal microbiota transplantation (FMT) is an attractive strategy to correct microbial dysbiosis in diarrhea-predominant irritable bowel syndrome (IBS-D). Although the mechanism of FMT is thought to be bacterial engraftment, the best approach to achieve engraftment after FMT in IBS-D (and other diseases) is not clear. We evaluated the effect of FMT (with or without pretreatment with antibiotics) on gut microbiome and symptoms in patients with IBS-D. In this randomized, placebo-controlled, single-center study, 44 patients with IBS-D with a least moderate severity (IBS severity scoring system, i.e., IBS-SSS, ≥175) were randomly assigned to one of four groups: single-dose oral FMT alone, single-dose oral FMT following a 7-day pretreatment course of Ciprofloxacin and Metronidazole (CM-FMT) or Rifaximin (R-FMT), or Placebo FMT. Primary endpoint was engraftment post-FMT and secondary endpoints were changes in IBS-SSS, and IBS-quality of life (IBS-QOL) at week 10. Median engraftment was significantly different among the three FMT groups (*P* = .013). Engraftment post-FMT was significantly higher in the FMT alone arm (15.5%) compared to that in R-FMT group (5%, *P* = .04) and CM-FMT group (2.4%, *P* = .002). The mean change in IBS-SSS and IBS-QOL from baseline were not significantly different among the four groups or between the three FMT groups combined vs. placebo at week 10. In summary, antibiotic pretreatment significantly reduced bacterial engraftment after FMT in patients with IBS-D.

## Introduction

Irritable Bowel Syndrome (IBS) is a chronic gastrointestinal condition which affects about 11% of the global population.^1^ IBS strongly impairs quality of life, work productivity and social function as well as inflicts substantial burden on health-care systems. IBS is characterized by abdominal pain associated with altered bowel habits and can be further categorized into diarrhea predominant (IBS-D), constipation predominant (IBS-C), or mixed subtype (IBS-M).^2^ Current IBS treatment options are limited and associated with poor patient satisfaction.^3,4^ This might be in part due to the fact that IBS is a heterogeneous disorder and treatments often do not target the underlying cause.^4^

The pathophysiology of IBS-D is complex and multifactorial involving dysfunction of gut-brain communication, altered intestinal permeability, mucosal immune activation, visceral hypersensitivity, altered gastrointestinal motility, and gut microbiota dysbiosis.^5^ The importance of gut microbiota dysbiosis in IBS is highlighted by the fact that triggers for IBS such as infections, poor sleep, antibiotic use, diet, and stress can affect intestinal microbiota composition.^6^ Indeed, studies have shown that gut microbial diversity is significantly reduced in IBS-D and composition of gut microbiota in IBS-D is different from healthy controls, including a decrease in genus *Faecalibacterium* and *Bifidobacterium*, and an increase in *Enterobacteriaceae* and *Bacteroides* among others.^7^ Using machine learning, investigators recently identified a potential microbial signature for severe IBS. Likewise, analyses of fecal metabolomes and microbiomes distinguished IBS from healthy controls suggesting that a microbial signature at the strain level may be present for IBS.^8^ In addition, transplantation of fecal microbiota from IBS-D individuals to germ-free mice resulted in alteration in gut function, immune activation and behavior in mice similar to that seen in IBS-D.^9^

A number of strategies to modulate gut microbiota in IBS have been proposed, including prebiotics, probiotics, dietary modifications, antibiotics, and recently fecal microbiota transplantation (FMT).^10^ To date, only a few randomized controlled trials (RCTs) have investigated the role of FMT in IBS, with conflicting results.^11–15^ The data on microbial engraftment after FMT in IBS is even more limited. Bacterial engraftment from FMT is thought to be one of the key factors responsible for the efficacy of FMT. However, bacterial engraftment after FMT appears to be complex and dependent on donor as well as recipient factors.^16,17^ A few studies have investigated the effect of FMT on microbiome in IBS, and none have specifically looked at bacterial engraftment after FMT.^13–15^ The best approach to achieve bacterial engraftment with FMT in IBS (as well as other diseases) is not clear and no study has evaluated the effect of pretreatment with antibiotics on bacterial engraftment after FMT. As antibiotics can modulate the gut microbiome, preconditioning with antibiotics such as oral vancomycin before FMT has been successfully used in multiple RCTs and observational studies to treat recurrent or refractory *Clostridioides difficile* infection.^18^ The primary objective of this study was to compare the effects of different antibiotic pretreatments on bacterial engraftment after FMT in patients with IBS-D. As this was a pilot randomized, placebo-controlled study designed to evaluate bacterial engraftment post-FMT in IBS-D with/without antibiotic pre-treatment, we acknowledge we were not adequately powered to assess clinical outcomes or bacterial engraftment.

## Results:

**Figure 1.**
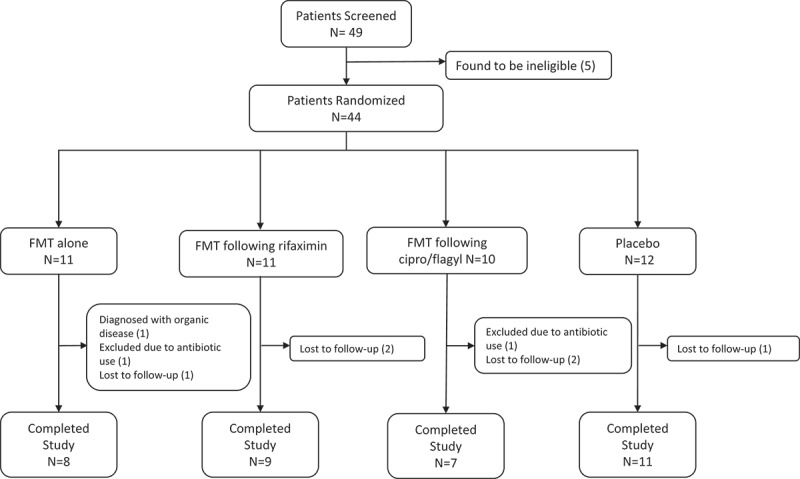
Study consort diagram.

Forty-four patients with IBS-D were randomized into the four arms – placebo, FMT alone, pretreatment with rifaximin 550 mg three times a day for 7 days followed by FMT (R-FMT), or pretreatment with ciprofloxacin 500 mg twice daily and metronidazole 500 mg three times a day for 7 days followed by FMT (CM-FMT) (Figure 1). One patient in the R-FMT arm was found to have microscopic colitis after completing the study and was not included in any study analyses. Demographic characteristics of the patients in the four arms were comparable (Table 1). However, there was a significant difference in IBS severity amongst the four arms at baseline (*P* = .03); the mean IBS severity as determined by IBS-SSS was the highest in the FMT alone arm (339.1) and the lowest in the R-FMT arm (282.3).

**Table 1.** Baseline characteristics of IBS patients in each of the four arms

Clinical Outcomes	FMT alone (n = 11)	R-FMT (n = 10)*	CM-FMT (n = 10)	Placebo (n = 12)
Female	6 (54.5%)	5 (50.0%)	6 (60.0%)	5 (41.7%)
Age (SD)	38.4 (11.5)	44.5 (18.4)	37.5 (13)	35.8 (14.2)
IBS-SSS (SD)	347.5 (59.0)	272.3 (53.4)	339.1 (85.4)	282.3 (70.7)
IBS-QoL (SD)	42.7 (19.2)	59.8 (19.3)	43.8 (23.0)	47.6 (13.4)

Note: Values are means (standard deviations). IBS-SSS = IBS-Severity Scoring System (range 0–500), lower value indicates lower symptom severity. IBS-QoL = IBS-Quality of Life (range 0–100), higher value indicates higher quality of life. *1 patient was not included in the baseline characteristic table due to being diagnosed with organic disease later (see consort table)

Two patients (one in the FMT alone arm and one in the placebo arm) developed a urinary tract infection or streptococcal pharyngitis requiring antibiotics between weeks 1 and 10. Thus, these 2 patients were not included in symptom or microbiome analyses at week 10. Six patients did not complete the clinical assessment or provide stool for microbiome analyses (i.e. were lost to follow-up) at week 10 (Figure 1). Of the remaining 35 patients, 3/35 patients did not provide a follow-up stool sample at week 10 and therefore were not included in the analysis for primary outcome, that is, bacterial engraftment. However, these three patients reported clinical outcomes at week 10 and were therefore included in analysis of secondary outcomes, that is, clinical outcomes at week 10.

## Effect of antibiotic pre-treatment on engraftment

**Figure 2.**
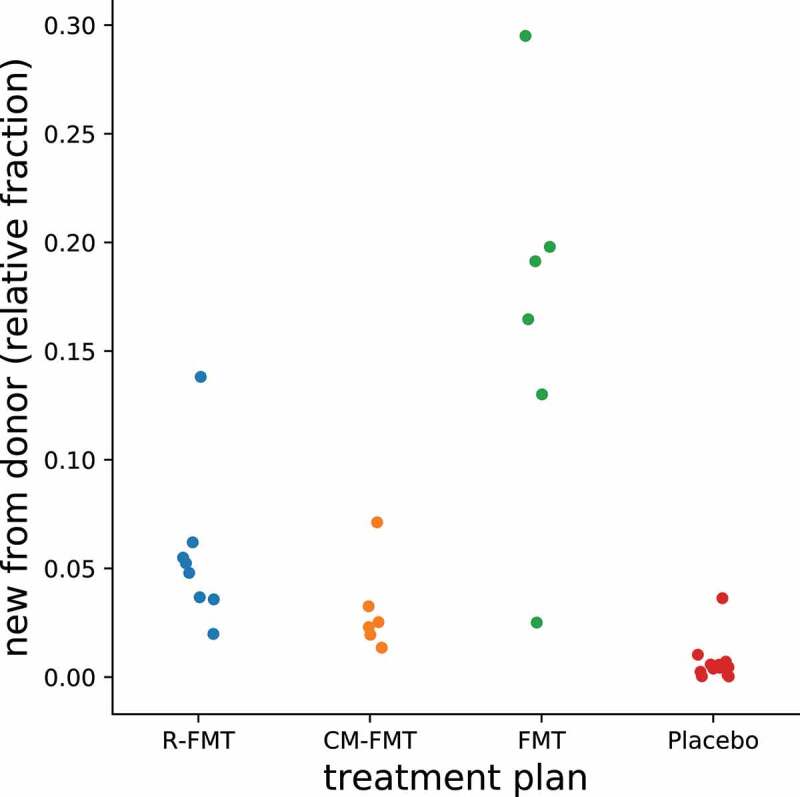
Effect of FMT (with and without antibiotic pretreatment) on bacterial engraftment averaged for week 1 and week 10 in patients with IBS-D.

Median engraftment rate was significantly different among the three FMT groups (*P* = .013). Median engraftment averaged for week 1 and week 10 was 15.5% in the FMT alone arm compared to 5% in R-FMT arm (*P* = .04) and 2.4% in CM-FMT arm (*P* = .002) (Figure 2). Higher median engraftment in the FMT alone arm was also observed for week 1 and week 10 separately compared to the other two FMT arms (Supplementary Figures 1, 2)

## Effect of antibiotics on microbial diversity and composition

**Figure 3.**
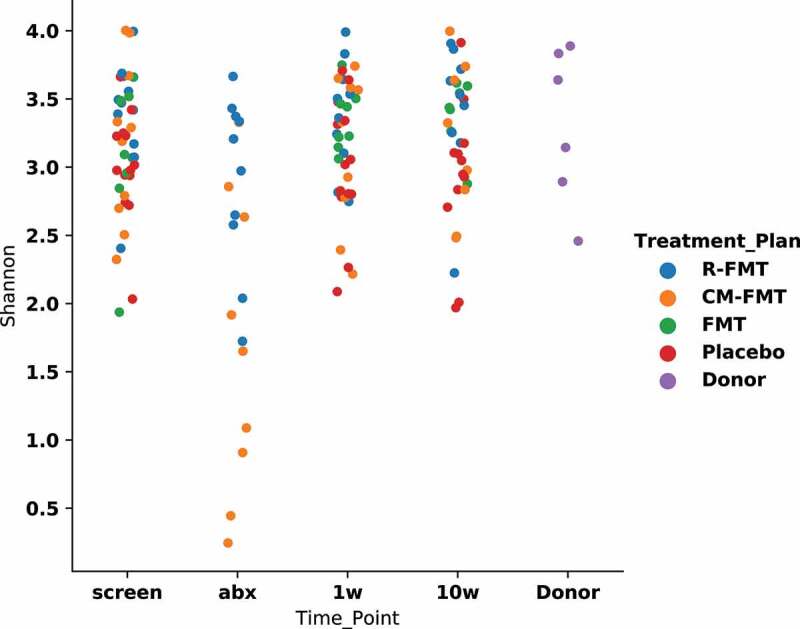
Effect of antibiotic pretreatment and FMT on alpha-diversity at various time-points in patients with IBS-D.

While alpha diversity was not different among IBS patients and heathy donors at baseline, alpha diversity was significantly reduced in the antibiotic arms during the antibiotic treatment (pre-FMT) compared to other time points (Figure 3) (*P* = .005 for R-FMT and *P* = .009 for CM-FMT group). Alpha-diversity at 1-week and 10-week was not significantly different from that at baseline for all the four groups.

**Figure 4.**
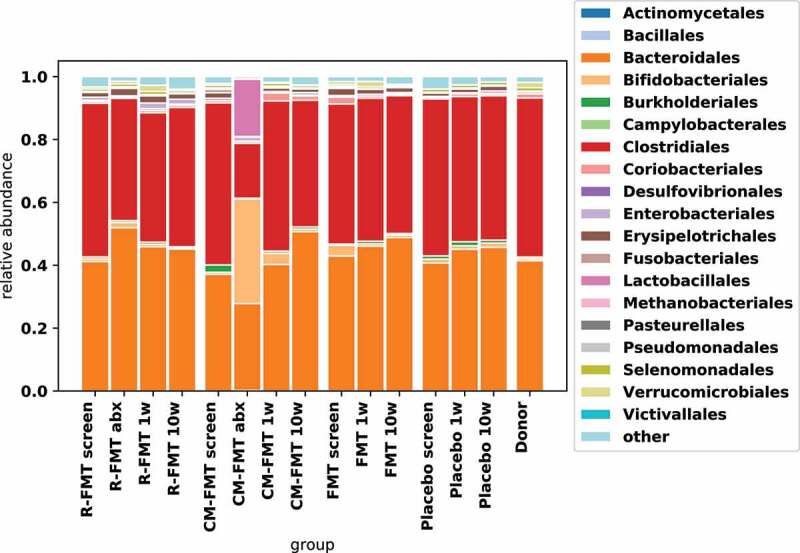
Effect of antibiotic pretreatment and FMT on microbial composition in IBS-D.

In CM-FMT arm, there was reduction in *bacteroidales* and increased abundance of gram-positive bacteria (*lactobacillales* and *bifidobacteriales*) during the antibiotic treatment (pre-FMT) (Figure 4). In R-FMT arm, pretreatment with rifaximin led to reduction in *Clostridiales* abundance during the antibiotic treatment (pre-FMT). No striking differences in microbial composition were noted 1-week and 10-week post-FMT/placebo vs. baseline in any of the four groups (Figure 4).

## Clinical outcomes at week 10

**Figure 5.**
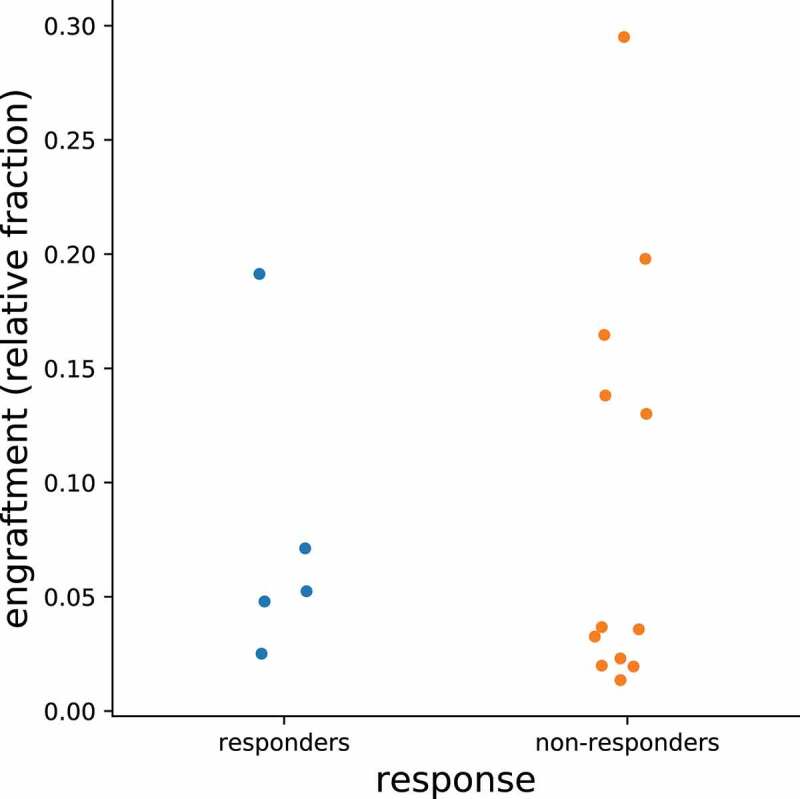
Engraftment rates averaged for week 1 and week 10 between IBS-D responders and non-responders.

Clinical outcomes (mean change in IBS-SSS and IBS-QoL, proportion of patients with adequate relief or global improvement) at week 10 were similar among the four arms (Table 2). Clinical outcomes at week 10 were also not different between the placebo arm and the three FMT arms combined (Table 3). These results were unchanged when adjusted for baseline IBS severity. Percent engraftment (averaged for week 1 and week 10) in the three FMT arms was not significantly different between clinical responders (decrease in IBS-SSS ≥ 50 points) and non-responders at week 10 (*P* = .75) (Figure 5). Percent engraftment calculated separately for week 1 or week 10 also did not differ between the clinical responders vs. non-responders (Supplementary Figures 3, 4). Baseline microbial composition also did not predict response status at week 10 (Supplementary Figure 5).

**Table 2.** Clinical outcomes among the four arms at week 10

Clinical Outcomes	FMT alone (n = 8)	R-FMT (n = 9)	CM-FMT (n = 7)	Placebo (n = 11)	p-value
Change in IBS-SSS	−32.3 (124.8)	−85.3 (94.6)	−114 (149.3)	−93.4 (97.1)	0.55
Change in IBS-QoL	15.4 (20.8)	19.3 (25.2)	−1.2 (7.6)	9.4 (18.4)	0.61
Number with adequate relief	2 (25%)	4 (44.4%)	4 (57.1%)	4 (36.4%)	0.66
Number with global improvement	2 (25%)	3 (33.3%)	2 (28.6%)	2 (18.2%)	0.95
IBS-SSS Responders	2 (25%)	5/8* (62.5%)	5 (71.4%)	7 (63.6%)	0.29
IBS-QoL Responders	2 (25%)	2 (22.2%)	5 (71.4%)	4 (36.4%)	0.21

Note: Change in score is the difference between week 10 and baseline. A negative value for change in IBS-SSS indicates an improvement in symptoms. A positive value for change in IBS-QoL indicates an improvement in quality of life. *1 patient did not complete IBS-SSS at week 10.

**Table 3.** Clinical outcomes between the placebo arm vs. all FMT arms combined at week 10

Clinical Outcomes	FMT arms (N = 24)	Placebo (n = 11)	P-value
Change in IBS-SSS	−75.6 (122.8)	−93.4 (97.1)	0.68
Change in IBS-QoL	14.5 (19.4)	9.4 (18.4)	0.47
Number with adequate relief	10 (41.7%)	4 (36.4%)	0.10
Number with global improvement	7 (29.2%)	2 (18.2%)	0.69
IBS-SSS Responders	12 (52.2%)	7 (63.6%)	0.72
IBS-QoL Responders	9 (37.5%)	4 (36.4%)	0.99

Note: Change in score is the difference between week 10 and baseline. A negative value for change in IBS-SSS indicates an improvement in symptoms. A positive value for change in IBS-QoL indicates an improvement in quality of life.

## Adverse events

Details of adverse events reported by patients is summarized in Table 4.

**Table 4.** Adverse events reported by study participants

FMT alone	R-FMT	CM-FMT	Placebo
Urinary tract infection (n = 1)	Nasal congestion (n = 1)	Brain fog (n = 1)	Streptococcal pharyngitis (n = 1)
		Severe headache (n = 1)	
		Genital HSV1 infection (n = 1)	

## Discussion

Our study found that antibiotic pre-treatment significantly reduced the bacterial engraftment after FMT suggesting that future FMT trials in IBS should not be preceded by antibiotics. Indeed, engraftment was highest in the group receiving FMT alone (i.e. without receiving antibiotics prior to FMT). Our study was not designed to be adequately powered to show improvement in symptoms though the results are worth reviewing. There was considerable improvement in IBS symptoms with FMT (with or without antibiotic pretreatment), however, this improvement was not statistically significantly different than placebo. Interestingly, bacterial engraftment with FMT did not impact symptom response in IBS-D.

Our results showing that engraftment was significantly higher in the arm receiving FMT alone is surprising given we had hypothesized that antibiotic treatment would enhance engraftment. In our study, FMT was administered 24 hours after finishing antibiotics. A previous study has shown that in addition to donor factors, bacterial engraftment after FMT is also modulated by recipient factors, i.e. abundance of OTUs in the patient are strongly correlated before and after FMT.^16^ It is possible that the effect of antibiotics before FMT significantly altered the host microbiome making it less receptive to the donor. Although ciprofloxacin and metronidazole have a half-life of 4–8 hours, it is possible that antibiotics (or their metabolites) had bactericidal effect on fecal transplant.^19–22^

We noted significant impact of antibiotics on microbiome in IBS-D patients. Rifaximin, which is an FDA approved treatment for IBS-D, reduced the relative abundance of *Clostridiales*, a finding that has been reported in previous studies.^23^ Fluoroquinolones, such as ciprofloxacin, have been shown to have significant bactericidal effects against gram-negative bacteria in fecal microbiome which was also seen in our patients.^24^ A decrease in gram-negative bacterial composition with ciprofloxacin was also accompanied with increase in abundance of gram-positive bacteria, such as *bifidobacteriales* and *lactobacillales*. Therefore, pre-treatment with antibiotic significantly changed the intestinal microbiome prior to FMT. These antibiotic-mediated microbiome changes were not as prominent at week 10 and 24 post-FMT. This is consistent with several studies that have shown that there is natural tendency of microbiome to return to pre-antibiotic treatment levels in a matter of days and weeks but some members are indefinitely lost.^25^ As antibiotic treatment always preceded FMT, how FMT changed this natural course is not clear.

We did not find any significant association between post-FMT bacterial engraftment with response to treatment. We also noted that engraftment was highest in FMT alone arm, but this arm did not have significantly different change in symptom severity compared to the other arms. Another study by Halkjaer *et al*. in IBS patients found that post-FMT microbiome resembled donor microbiota more closely than pre-FMT microbiome.^13^ Similar to our study, they also found that long-term establishment of donor microbial species with FMT in IBS was not sufficient to improve the clinical outcomes of patients.^13^ On the other hand, Goll et al recently reported that responders to FMT had a trend of convergence toward donor microbiome profile after FMT while this trend was less clear for non-responders.^26^El-Salhy *et al* did not find any improvement in ‘dysbiosis index’ with FMT in IBS patients despite finding FMT was effective in improving IBS symptoms.^14^ Our study was designed to detect the effect of antibiotic pretreatment on post-FMT bacterial engraftment and not the association between bacterial engraftment and clinical response. Thus, more studies are needed to assess the role of engraftment in symptom response in IBS-D and elucidate the host and donor microbiome factors associated with clinical response.

Although we were not powered to study post-FMT clinical outcomes, FMT with/without antibiotic pre-treatment was clinically not superior to placebo in our study. This is in agreement with Aroniadis *et al*. who also did not find any improvement in IBS symptoms with FMT oral capsules in patients with IBS-D.^12^ It is also possible that oral FMT capsule is not an effective treatment for IBS, as a recent meta-analysis found that oral FMT capsules were inferior to placebo capsules in two pooled trials.^27^ However, it is possible that the high placebo response of over 60% in our study population made it difficult to detect the possible benefit of FMT within the constraints of our sample size. Other factors that have been shown to be associated with FMT response in IBS include colonoscopy or nasojejunal guided FMT administration, use of “super-donor” and post-infectious IBS subgroup.^12,14,15,27^ Future studies should delineate the role of FMT in IBS-D.

Our study had several limitations. First, as this study was based on feasibility, no formal sample size calculation was performed, and we did not enroll the planned number of participants. This might have led to potential under-powering of the study and not being able to detect differences in bacterial engraftment and/or clinical outcomes among the four groups. However, even with this small sample size we were able to show significant differences in our primary outcome (post-FMT bacterial engraftment) with our current sample size. Moreover, the study was not designed to study the clinical efficacy of FMT (with/without antibiotic pretreatment) in IBS-D. Second, although 44 patients were randomized, data from 9 patients (26.4%) could not be included because of various reasons detailed in Figure 1. Third, although investigators did not disclose this to participants unless directly asked, it is possible that patients randomized to antibiotic groups were not ‘blinded’ to treatment as there was no antibiotic pre-treatment in the placebo arm. Fourth, we understand that post-FMT bacterial engraftment can be influenced by a variety of host and environmental factors which were not controlled for in this study.

Despite these limitations, our study had several strengths. This is the first study evaluating the effect of antibiotic pretreatment on FMT engraftment in IBS. Knowledge and concepts about the effect of antibiotic pre-treatment on bacterial engraftment will be informative for future FMT trials in IBS as well as other diseases. Second, we observed that bacterial engraftment with FMT did not translate into symptom improvement. Thus, the lack of benefit with oral FMT capsule in IBS does not appear to be related to lack of engraftment. In addition, future studies investigating the role of FMT in IBS via other routes should investigate the underlying mechanisms of the benefit with FMT (if any).

In summary, antibiotic pretreatment significantly reduced bacterial engraftment after FMT. FMT (with or without antibiotic pretreatment) was not superior to placebo in improving gastrointestinal symptoms in IBS-D. Bacterial engraftment with FMT did not translate into symptom improvement in IBS-D.

## Patients and methods:

### Trial design

Participants were included in a single-centered, placebo-controlled pilot study and randomly allocated (1:1:1:1) to one of four treatment arms: placebo, FMT alone, pretreatment with rifaximin 550 mg three times a day for 7 days followed by FMT (R-FMT), or pretreatment with ciprofloxacin 500 mg twice daily and metronidazole 500 mg three times a day for 7 days followed by FMT (CM-FMT). Antibiotics were discontinued at least 24 hours prior to receiving FMT. For 2 days prior to FMT, participants were treated with omeprazole 20 mg BID. Immediately after finishing the last antibiotic dose, a bowel cleanse (magnesium citrate, up to 3 bottles) was administered 24 hours prior to FMT.

Patients were seen for baseline (screening), treatment (FMT or placebo capsules), 1 and 10 weeks post treatment. Patients completed the following questionnaires at the screening visit, weeks 1 and 10 post FMT: IBS-severity scoring system (IBS-SSS), IBS-specific quality of life (IBS-QoL), IBS global improvement scale (IBS-GIS), and adequate relief of IBS symptoms. Additionally, patients were asked to provide stool and blood at baseline, week 1, and week 10. They were assessed for any adverse events throughout the study. Written informed consent was obtained from each patient and the study was approved by the Committee on Clinical Investigations at Beth Israel Deaconess Medical Center.

### End points

The primary objective of this pilot study was to compare the stable engraftment of a donor’s microflora to a recipient with IBS-D after FMT (with or without pretreatment with antibiotics) to placebo. Secondarily, we evaluated change in IBS-SSS, IBS-QoL, IBS-GIS, and proportion of patients reporting adequate relief of symptoms at week 1 and 10 among the four arms.

### Patients

We enrolled patients who were between 18 and 80 years of age and who had IBS-D (Rome III criteria) between July 2016 and April 2018. Enrolled patients were included if they had active IBS symptoms at screening (as defined by having IBS-SSS score >150), a colonoscopy with normal random biopsies following the onset of IBS symptoms and within five years or since the onset of any of the following alarm features (if applicable): unintentional weight loss, nocturnal symptoms, and rectal bleeding or anemia. Patients were allowed to stay on their IBS medications provided they had been on a stable dose for at least 30 days prior to entering the study and were not planning to change the dose or make changes to their diet or lifestyle.

Exclusion criteria included i) patients with organic disease of their GI tract such as inflammatory bowel disease, pancreatitis, or malignancy; ii) patients who had major abdominal surgery *excluding* cholecystectomy (as long as the IBS symptoms predated the surgery and there was no evidence of post-cholecystectomy biliary tract pain) appendectomy, polyp removal, hysterectomy, tubal ligation, C-section); iii) patients with recent use of antibiotics within 28 days; iv) patients with immunodeficiency or intolerant of/or hypersensitive to ciprofloxacin, metronidazole, or rifaximin.

### Intervention

FMT consisted of a single dose of 19 capsules with each pill consisting of 0.75 g of frozen fecal filtrate (OpenBiome, Somerville, Ma). After visual inspection and weighing, stool was transferred under aerobic conditions to a sterile 330 μm filter bag, diluted in a sterile, US Pharmacopoeia-grade glycerol saline solution (12.5% glycerol in 0.90% w/v NaCl in water), and fully homogenized while still in the filter bag using a paddle blender for at least 180 seconds. During this process, fibrous material remains on one side of the filter while bacteria, small molecules, and water are pressed to the other side of the filter. Each FMT preparation was derived using filtrate from a single donor; filtrates from different donors were never mixed. Capsules were made from six donors and same six donors were used for all three groups. All three groups received FMT from six donors. Placebo consisted of 19 capsules containing glycerol with brown coloring agent. FMT or placebo was administered after undergoing magnesium citrate-based bowel cleanse the day before. FMT or placebo capsules were taken under direct supervision at the Division of Gastroenterology at Beth Israel Deaconess Medical Center, Boston, USA, where the study visits took place. The identity of the capsules was unknown to participants, researchers and primary investigators.

### Fecal sample collection

Fecal samples were collected from patients at baseline before bowel cleansing, during antibiotic treatment period (3 days before FMT treatment), 1-week and 10-week post treatment. The collected fresh feces were stored frozen in RNA later until the completion of study

### Measures

#### IBS-SSS:

This is a validated scale for assessing overall IBS symptom severity (ranging from 0 to 500) with higher scores suggesting higher symptom severity.^28^ A patient was considered an IBS-SSS responder if he/she had at least a 50-point decrease in their IBS-SSS from baseline at the 10-week follow-up.

#### IBS-QoL:

This is a 34-item assessment of the degree to which the condition interferes with a patient’s quality of life. Each item is rated on a five-point Likert scale and a linear transformation yields a summed score with a theoretical range of 0 to 100, a higher score indicating better quality of life.^22^ A patient was considered responder if he/she had at least a 12-point improvement in their IBS-QoL from baseline at the 10-week follow-up.

#### Global assessment of improvement:

Patients were asked “Compared to the way you felt before you entered the study, have your IBS symptoms over the past 7 days been: (1) = substantially worse, (2) = moderately worse, (3) = slightly worse, (4) = no change, (5) = slightly improved, (6) = moderately improved, or (7) = substantially improved.^23,24^ A patient reporting moderate or substantial improvement in symptoms was considered a responder.

#### Adequate relief:

Our other main outcome was adequate relief, which is a single dichotomous categorization that asks participants “Over the past week have you had adequate relief of your IBS symptoms?” A patient who reports having adequate relief at follow-up was considered a responder.

## 16s sequencing

Stool samples were shipped in RNA later to the Broad Institute at the Massachusetts Institute of Technology for 16S rRNA sequencing. After washing away RNA later 2x with PBS, the Qiagen DNeasy PowerSoil–htp 96 Well Soil DNA Isolation Kit (MO BIO Laboratories) was used with bead beating on a TissueLyzer II at 20 Hz for 10 min as per manufacturer’s protocol. PCR amplifcation of the V4 region of 16S rRNA gene, and Illumina paired-end sequencing was performed, as described previously.^29^

16S Processing Primers were trimmed, paired ends merged, and operational taxonomic units (OTUs) identified with a custom pipeline. OTUs were grouped into 97% identical clusters. OTUs represented in fewer than two unique samples, and OTUs with fewer than 10 reads were discarded. Taxonomic assignments for each OTU were called using the RDP database.

Microbial Community Analysis: For alpha-diversity calculations, the Shannon diversity index was calculated for each sequenced stool sample. All samples had over 10,000 reads except for one sample taken from a patient immediately after antibiotics, which had only 843. All 16S rRNA raw data was uploaded to Zenodo and is publicly available (https://zenodo.org/record/5218850).

## Engraftment

For each patient, engraftment was defined based on the presence of OTUs in both the FMT donor and the patient’s post-FMT stool sample, as well as absence in the patient’s pre-FMT stool sample (collected at baseline prior to antibiotics). For the primary outcome, engraftment was calculated as average of 1-week and 10-week post-FMT engraftments. In addition, engraftment was also calculated and reported separately for week 1 and week 10. Median engraftment among the three FMT groups were compared using the Kruskal–Wallis with Dunn’s posttest. Paired t-tests were used to compare diversity before and after FMT, and independent t-tests or Mann–Whitney U-tests were used for other comparisons; all reported *p*-values are two-sided.

## Statistical analysis plan

All statistical analyses were undertaken on a per protocol basis, using two-tailed tests with alpha set at 5%. For the continuous outcome measures (i.e. IBS-SSS and IBS-QOL), we used analysis of variance (ANOVA) to compare the four treatment arms on magnitude of change from baseline to the 10-week endpoint. If the omnibus ANOVA for either of these outcome measures was significant, we planned to conduct pairwise comparisons using Tukey’s HSD tests. Given that this was a pilot study, and we anticipated relatively small sample sizes, we also planned to conduct independent samples t-tests comparing improvement in the three active FMT arms *combined* against improvement in the placebo FMT arm to improve statistical power.

For dichotomous outcome measures, we planned to use Fisher’s exact test, which provides more accurate *p*-values as compared to the chi-squared test when sample sizes are small. If any omnibus test was significant, we planned to follow-up with Fisher’s exact tests to examine all pairwise comparisons. Similar to the analyses for the continuous measures (and for the same reasons), we also planned to use Fisher’s exact test to compare the dichotomous outcomes in the three active FMT arms *combined* against the dichotomous outcomes in the placebo FMT arm.

We are mindful that our analysis plan does not control for multiple comparisons. However, given the fact that this was a pilot study with relatively small sample sizes, we chose to risk Type II errors (but to note this potential for inflated Type II error as we have here), as opposed to apply stringent controls on the Type I error rate, which would make it virtually impossible to detect any potential effects unless they were of enormous size.

## Sample size

Given this was a pilot study, we planned to enroll 80 patients in the study based on feasibility. However, during the enrollment period, the capsules used to encapsulate the FMT were no longer being used by OpenBiome and enrollment was ended early. Therefore, 44 patients were randomized into this study.

## Supplementary Material

Supplemental Material

## Data Availability

All 16S rRNA raw data was uploaded to Zenodo and is publicly available (https://zenodo.org/record/5218850). The clinical data that support the findings of this study are available from the corresponding author, PS, upon reasonable request.

## Trial registration

ClinicalTrials.gov NCT02847481 https://clinicaltrials.gov/ct2/show/NCT02847481
