# Supplementary figures and images for: Effect of antibiotic pretreatment on bacterial engraftment after Fecal Microbiota Transplant (FMT) in IBS-D
Source: Gut Microbes. 2022 Jan 11;14(1):2020067. doi: 10.1080/19490976.2021.2020067 (PMC8757476; doi:10.1080/19490976.2021.2020067)

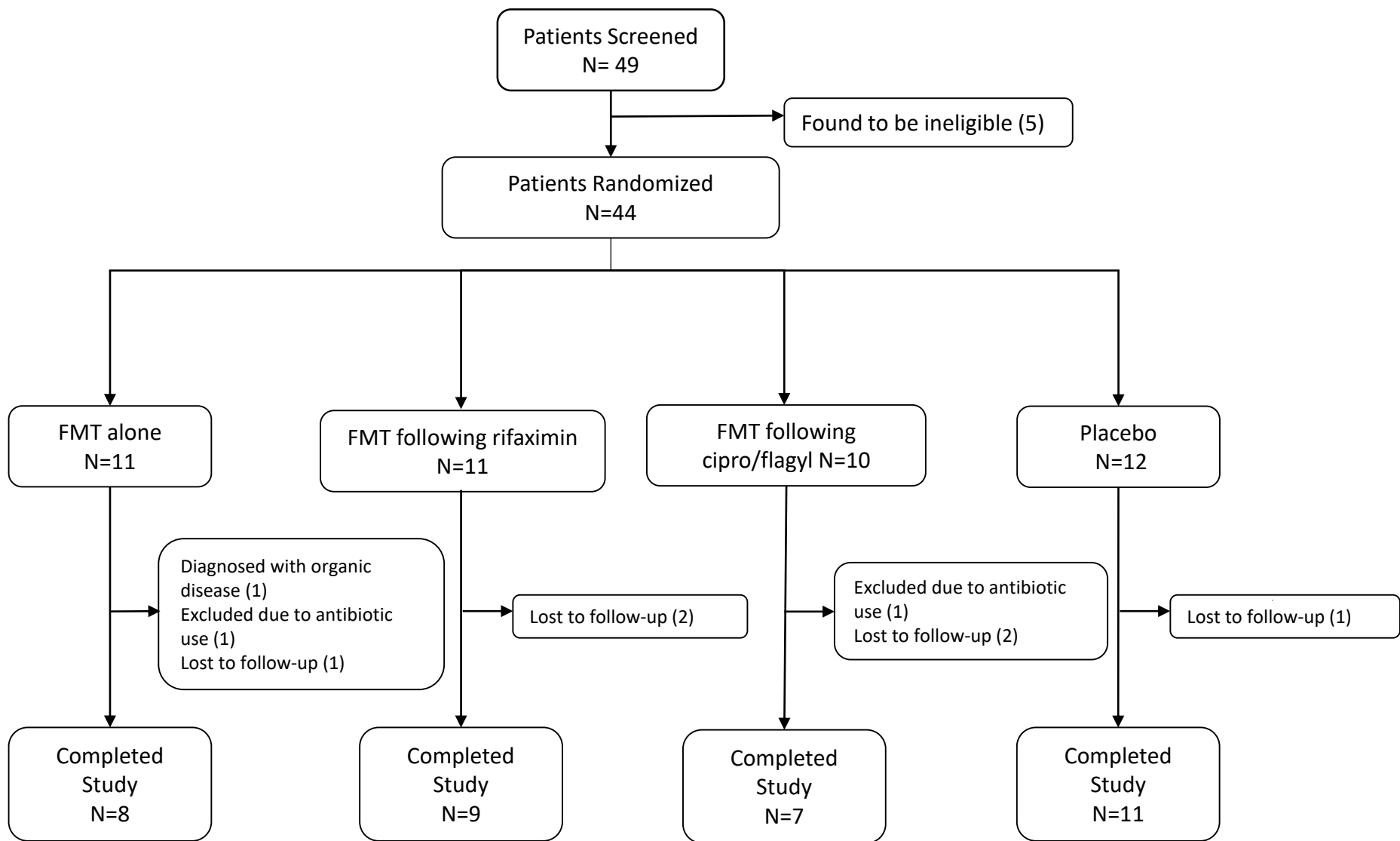

Supplement: Supplemental Material [file KGMI_A_2020067_SM2039.zip › supplementary/sup fig 1.pdf]

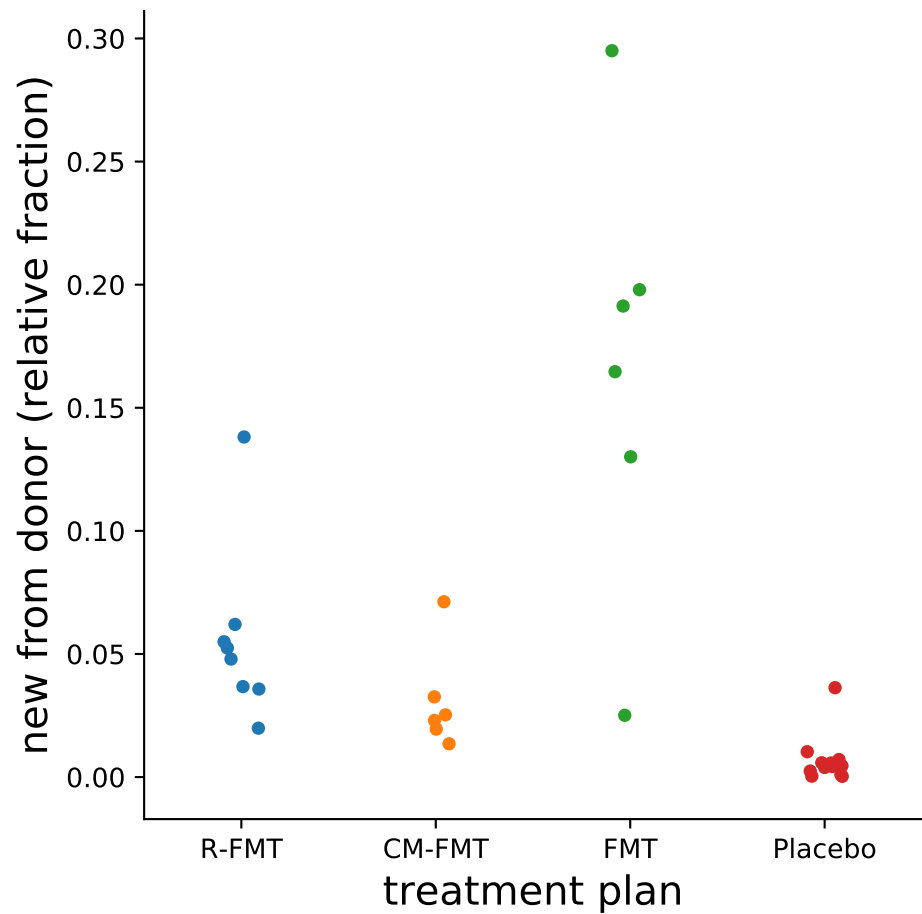

Supplement: Supplemental Material [file KGMI_A_2020067_SM2039.zip › supplementary/sup fig 2.pdf]

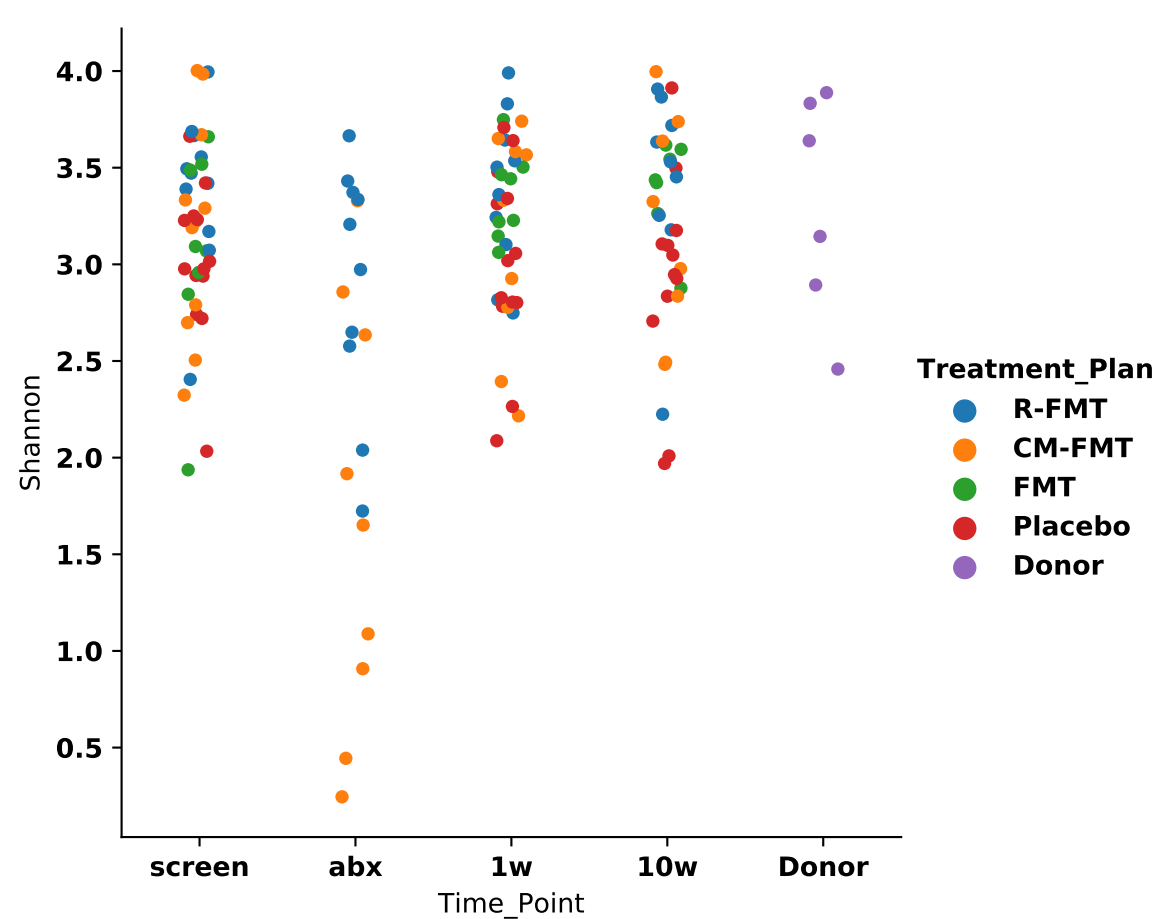

Supplement: Supplemental Material [file KGMI_A_2020067_SM2039.zip › supplementary/sup fig 3.pdf]

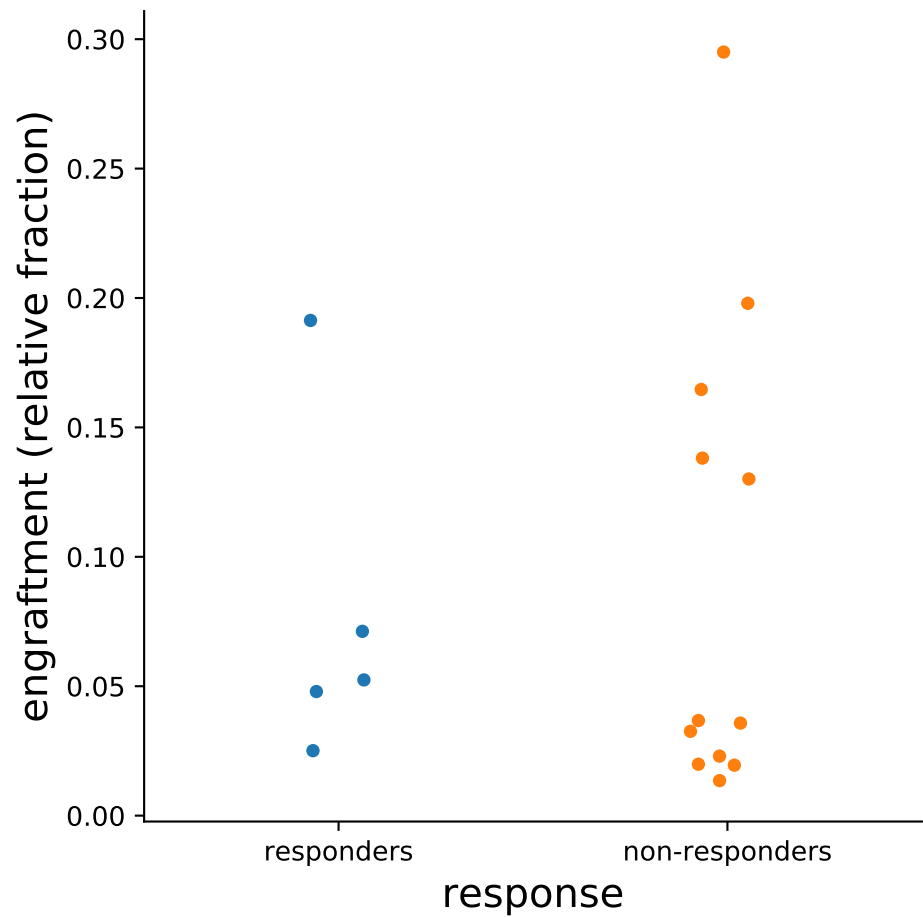

Supplement: Supplemental Material [file KGMI_A_2020067_SM2039.zip › supplementary/sup fig 5.pdf]
