# Supplementary material for: Effect of antibiotic pretreatment on bacterial engraftment after Fecal Microbiota Transplant (FMT) in IBS-D
Source: Gut Microbes. 2022 Jan 11;14(1):2020067. doi: 10.1080/19490976.2021.2020067 (PMC8757476; doi:10.1080/19490976.2021.2020067)
Supplement: Supplemental Material [file sup fig 4.pdf › supplementary/sup fig 4.pdf]

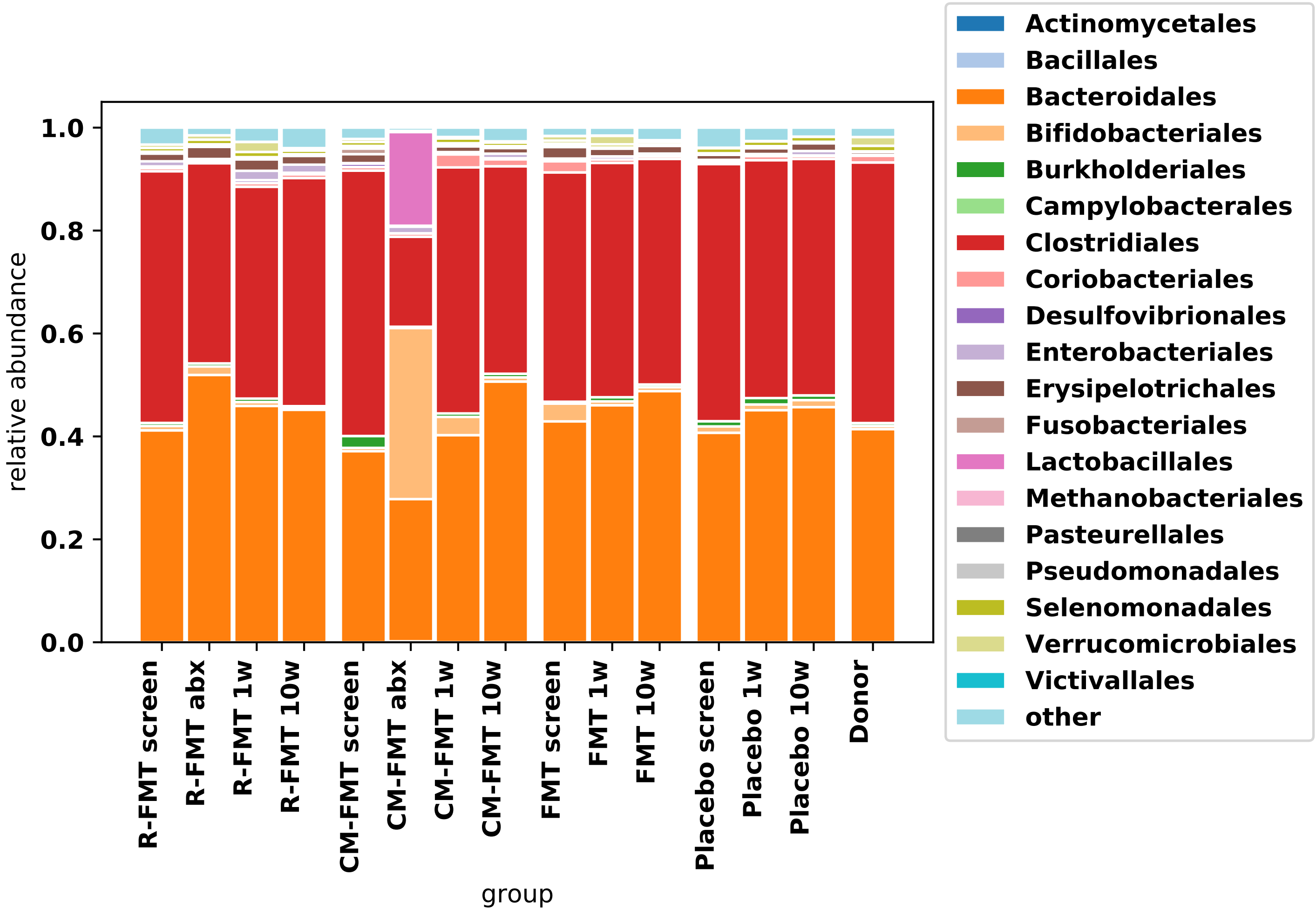
relative abundance
0.0
0.2
0.4
0.6
0.8
1.0
group
R-FMT screen
R-FMT abx
R-FMT 1w
R-FMT 10w
CM-FMT screen
CM-FMT abx
CM-FMT 1w
CM-FMT 10w
FMT screen
FMT 1w
FMT 10w
Placebo screen
Placebo 1w
Placebo 10w
Donor
Actinomycetales
Bacillales
Bacteroidales
Bifidobacteriales
Burkholderiales
Campylobacterales
Clostridiales
Coriobacteriales
Desulfovibrionales
Enterobacteriales
Erysipelotrichales
Fusobacteriales
Lactobacillales
Methanobacteriales
Pasteurellales
Pseudomonadales
Selenomonadales
Verrucomicrobiales
Victivallales
other
